# Plasma Amino Acid Concentrations Predict Mortality in Patients with End-Stage Liver Disease

**DOI:** 10.1371/journal.pone.0159205

**Published:** 2016-07-13

**Authors:** Benedict Kinny-Köster, Michael Bartels, Susen Becker, Markus Scholz, Joachim Thiery, Uta Ceglarek, Thorsten Kaiser

**Affiliations:** 1 Institute of Laboratory Medicine, Clinical Chemistry and Molecular Diagnostics, University Hospital Leipzig, Leipzig, Germany; 2 Department of Visceral, Vascular, Thoracic and Transplant Surgery, University Hospital Leipzig, Leipzig, Germany; 3 LIFE – Leipzig Research Center for Civilization Diseases, University Hospital Leipzig, Leipzig, Germany; 4 Institute for Medical Informatics, Statistics and Epidemiology, University of Leipzig, Leipzig, Germany; Chiba University, Graduate School of Medicine, JAPAN

## Abstract

**Background:**

The liver plays a key role in amino acid metabolism. In former studies, a ratio between branched-chain and aromatic amino acids (Fischer’s ratio) revealed associations with hepatic encephalopathy. Furthermore, low concentrations of branched-chain amino acids were linked to sarcopenia in literature. Encephalopathy and sarcopenia are known to dramatically worsen the prognosis. Aim of this study was to investigate a complex panel of plasma amino acids in the context of mortality in patients with end-stage liver disease.

**Methods:**

166 patients evaluated for orthotopic liver transplantation were included. 19 amino acids were measured from citrated plasma samples using mass spectrometry. We performed survival analysis for plasma amino acid constellations and examined the relationship to established mortality predictors.

**Results:**

33/166 (19.9%) patients died during follow-up. Lower values of valine (p<0.001), Fischer’s ratio (p<0.001) and valine to phenylalanine ratio (p<0.001) and higher values of phenylalanine (p<0.05) and tyrosine (p<0.05) were significantly associated with mortality. When divided in three groups, the tertiles discriminated cumulative survival for valine (p = 0.016), phenylalanine (p = 0.024) and in particular for valine to phenylalanine ratio (p = 0.003) and Fischer’s ratio (p = 0.005). Parameters were also significantly correlated with MELD and MELD-Na score.

**Conclusions:**

Amino acids in plasma are valuable biomarkers to determine increased risk of mortality in patients with end-stage liver disease. In particular, valine concentrations and constellations composed of branched-chain and aromatic amino acids were strongly associated with prognosis. Due to their pathophysiological importance, the identified amino acids could be used to examine individual dietary recommendations to serve as potential therapeutic targets.

## Introduction

The human liver is crucial to metabolic processes. Imbalances in amino acid metabolism and alterations of amino acid levels in human blood due to liver diseases are described in existing literature [1,2]. Already in 1971, Fischer et al. presented data indicating that low levels of the ratio between the structurally related branched-chain amino acids (BCAA) valine, leucine and isoleucine and the structurally related aromatic amino acids (AAA) phenylalanine, tyrosine and tryptophan (BCAA / AAA, Fischer’s ratio) promote hepatic encephalopathy in cirrhotic patients [3,4]. Changes in amino acid levels in blood affect a large number of different metabolic pathways, but to our knowledge until now there is no study available investigating the complex relationship between plasma amino acid concentrations and prognosis in patients with end-stage liver disease.

In the context of liver transplantation, prognosis in patients with liver cirrhosis can be estimated as three-month mortality risk by using the MELD (Model for End-Stage Liver Disease) score [5,6]. For many patients with end-stage liver disease, the only curative therapy is an orthotopic liver transplantation (OLT). The MELD scoring system has replaced the Child-Pugh-Turcotte based classification for listing in UNOS (United Network of Organ Sharing) area in 2002. It was introduced into the Eurotransplant community in 2006. Lab MELD score is calculated with an algorithm consisting of the three laboratory parameters serum creatinine, serum bilirubin and INR (International Normalized Ratio) [7].

The MELD score is well evaluated and widely accepted as an objective predictor [8]. Although waiting list mortality has decreased since MELD implementation [9], former studies revealed that mortality might not be predicted appropriately by MELD score in patients suffering from frequent complications of cirrhosis. Complications such as hepatic encephalopathy [10], sarcopenia [11], malnutrition [12], infections [13], esophageal bleeding [14] and hyponatremia [15] have a substantial impact on survival. Some promising advancements e.g. MELD-Na score were presented in the last years, but did not find a use in clinical routine yet [16,17].

The aim of this study was the evaluation of the prognostic value of a complex panel of amino acid concentrations in human blood plasma for predicting mortality. We analyzed 19 metabolites including all essential amino acids in a collective of patients evaluated for an OLT.

## Materials and Methods

### Study population

Patients were recruited within the evaluation process for liver transplantation at the University Hospital of Leipzig. The Ethics Committee at the Leipzig University Faculty of Medicine approved for the retrospective usage of residual material and data for our study.

Our collected cohort consisted of 231 plasma samples from 173 different patients. For some patients, samples from more than one date were available because of the reevaluation guidelines of the German Medical Association. In these cases, only the patient sample from the first blood withdrawal was used for analysis. The cohort was not necessarily at fasting before blood taking. 7 patients were excluded for the study due to renal replacement therapy (N = 3) or anticoagulation therapy with phenprocoumon (N = 3) or rivaroxaban (N = 1), so our analyzed study population consisted of 166 samples from different patients. Survival data and patient characteristics were collected from the patient information system and from administration data from the University Hospital’s transplant office. All causes of mortality were considered as deceased (N = 33, 19.9%). For patients who were transplanted during follow-up period (N = 18; 10.8%), follow-up ended at date of transplantation and patients were censored at that time for survival analysis. Median follow-up time in the non-transplanted surviving cohort (N = 115; 69.3%) was 22.0 months (range 8.8 to 24.0 months). Baseline characteristics are presented in Tables 1 and 2.

**Table 1:** Baseline characteristics in the analyzed population.

	Female (N = 59), Median	Male (N = 107), Median	Total (N = 166), Median	Range
Age [years]	55.98	58.62	57.72	20.36–76.96
MELD score	10.99	11.43	11.42	6.43–39.63
MELD-Na score	13.78 (N = 58)	14.13 (N = 105)	14.00 (N = 163)	6.00–40.00
Creatinine [μmol/l]	70	82	78.5	35–313
Bilirubin [μmol/l]	27.7	27.9	27.85	4.4–346.6
INR	1.3	1.2	1.3	0.9–4.3
Sodium [mmol/l]	137.9 (N = 58)	138.0	138.0, N = 163	117.0–151.0

**Table 2:** Description of liver disease etiologies, comorbidities and Child-Pugh-Turcotte classification in numbers and percentage in the analyzed population (N = 166).

**Etiology of liver cirrhosis**	**N**	**%**
Nutritive-ethyltoxic	108	65.1
Viral hepatitis	12	7.2
Autoimmunehepatitis	5	3.0
PSC	4	2.4
Others	9	5.4
Cryptogenic	17	10.2
Undefined	11	6.6
**Comorbidities**		
HCC	22	13.3
Diabetes mellitus	53	31.9
**Child-Pugh-Turcotte**		
A	59	35.5
B	53	31.9
C	21	12.7
undefined	33	19.9

PSC: Primary sclerosing cholangitis. HCC: Hepatocellular Carcinoma.

### Amino acids analysis

Measurement and preanalytical standardization of the metabolites was performed according to published protocols [18,19]. Briefly, citrated plasma samples were diluted 1:10 with methanol and centrifugated at 13000U/min for 10 minutes at room temperature. 100 μl of the internal standard solution was added to 10 μl of the supernatant in 96-well polypropylene microtiter plates. After butanolic esterification, samples were analyzed by liquid chromatography tandem-mass spectrometry (API 2000, Sciex, Darmstadt, Germany). Each analytical batch contained two quality control samples. Mean inter-assay coefficient of variation was below 14.2%. We used ChemoViewTM 1.4.2 software (Sciex, Darmstadt, Germany) for quantification of the measured panel.

### Laboratory data

Residual material of citrated plasma was aliquoted after registration in the medical laboratory and stored at -80°C. The panel measured by mass spectrometry included 19 different amino acids. Values were multiplied with a factor of 10/9 according to 1:10 dilution with trisodium citrate solution at blood drawing (S-Monovette^®^ 3ml 9NC, Sarstedt, Nümbrecht, Germany). Plasma concentrations are presented in μmol per liter. Leucine and isoleucine could not be distinguished by the method. Due to technically invalid measurement, two concentrations of methionine were discarded.

The amount of branched-chain amino acids (BCAA) was calculated as sum of valine, leucine and isoleucine. The concentration of aromatic amino acids (AAA) was determined as sum of phenylalanine, tyrosine and tryptophan. Fischer’s ratio is defined as BCAA to AAA ratio. We also calculated the BCAA to tyrosine ratio (BTR) and valine to phenylalanine ratio (VPR) for analysis.

Clinical chemistry including MELD score components creatinine (serum, enzymatic assay creatinine Plus Ver. 2, Roche, Mannheim, Germany), bilirubin (serum, Total DPD Gen.2 kit, Roche, Mannheim, Germany) and INR (International Normalized Ratio, plasma, ACL TOP 700 System, Instrumentation Laboratory, Lexington, USA) was measured due to the clinical request at University Hospital of Leipzig for each patient. For 163 of 166 patients, serum sodium concentrations were already available from clinical routine. Clinical parameters examined for this study were analyzed retrospectively.

MELD score was calculated according to the guidelines of the UNOS [20] using the following formula:

MELD score = 10 * (0.957 * ln(creatinine [mg/dl]) + 0.378 * ln(bilirubin [mg/dl]) + 1.12 * ln(INR) + 0.643).

MELD-Na score was calculated according to Kim et al. [16]:

MELD-Na score = MELD–Na [mmol/l]–(0.025 * MELD * (140 –Na [mmol/l])) + 140.

### Statistics

Calculation was performed using SPSS 20 (SPSS Inc., Chicago, IL, USA). Amino acid data were transformed using natural logarithm or area sinus hyperbolicus function for approximation of Gaussian distributions. Survival analysis was performed with Cox Proportional Hazards Regression Model. For Log-Rank testing, we divided our study cohort in three groups dependent on tertiles (T1: below the 33^rd^ percentile; T2: within the 33^rd^ and 67^th^ percentile; T3: above the 67^th^ percentile). Mann-Whitney-U-Test was used for analysis between groups when divided in the deceased and surviving cohort or in three different MELD ranges (low scores: 6–9, moderate scores: 10–19, high scores: 20–40), respectively. Correlation analysis was performed using Spearman’s rank correlation coefficient. Binary logistic regression analysis was performed to analyze the predictive value of the biomarkers for mortality in addition to MELD score. P-values below 0.05 were considered as statistically significant. In detail, levels of significance are presented as * = p<0.05, ** = p<0.01 and *** = p<0.001. A correction for multiple testing was not performed.

## Results

### Survival analysis

In our study population of 166 patients, 33 patients (19.9%) died during follow-up period without receiving a liver transplant.

To examine the relationship between survival and plasma concentrations of the measured metabolites, data were analyzed using the Cox-Regression model (Table 3). We identified a significantly higher mortality rate for higher values of the amino acids phenylalanine (p<0.05) and tyrosine (p<0.05). In contrast, lower valine plasma concentrations were significantly related to a higher mortality rate (p<0.001). Lower values of the amino acid constellations Fischer’s ratio (p<0.001), BCAA to tyrosine ratio (BTR, p<0.01) and valine to phenylalanine ratio (VPR, p<0.001) were all negatively associated with survival.

**Table 3:** Data of the measured metabolite panel (N = 166). Survival analysis was performed by an univariate cox-regression model. Significant results are written in bold and asterisked (*, **, ***). Essential amino acids are printed in italics.

	Range	β-cox	p-value
Alanine	104.07–610.11μmol/l	-0.40	0.451
Arginine	8.70–239.00μmol/l	0.44	0.337
Aspartic acid	0.87–117.33μmol/l	0.51	0.093
Citrulline	9.80–114.23μmol/l	0.36	0.370
Glutamic acid	67.03–653.38μmol/l	-0.90	0.095
Glycine	102.44–524.70μmol/l	1.42	0.054
Histidine	27.97–186.07μmol/l	0.85	0.153
*Leucine* and *Isoleucine*	54.07–414.20μmol/l	-0.39	0.494
*Lysine*	51.45–524.37μmol/l	-0.23	0.746
*Methionine* (N = 164)	10.49–658.97μmol/l	0.51	0.053
Ornithine	23.80–420.17μmol/l	0.37	0.362
*Phenylalanine*	27.20–276.09μmol/l	**1.10**	0.018*
Proline	87.77–364.23μmol/l	0.37	0.523
Serine	43.07–817.60μmol/l	-0.66	0.118
*Threonine*	8.33–61.13μmol/l	-0.22	0.656
*Tryptophan*	4.37–32.81μmol/l	-0.02	0.957
Tyrosine	31.47–235.49μmol/l	**0.91**	0.047*
*Valine*	71.70–365.60μmol/l	**-2.47**	<0.001***
BCAA	157.00–779.80μmol/l	**-1.53**	0.030*
AAA	81.40–538.26μmol/l	**1.15**	0.024*
Fischer’s ratio (BCAA / AAA)	0.56–3.95	**-2.17**	<0.001***
BTR (BCAA / Tyr)	0.98–9.37	**-1.42**	0.004**
VPR (Val / Phe)	0.62–5.68	**-2.27**	<0.001***

Val: Valine. Phe: Phenylalanine. Tyr: Tyrosine.

BCAA: Branched-chain amino acids, sum of valine, leucine and isoleucine.

AAA: Aromatic amino acids, sum of phenylalanine, tyrosine and tryptophan.

We compared data between the deceased and surviving cohort for these parameters in Table 4.

**Table 4:** Comparison of distributions between the deceased and surviving patients was performed using Mann-Whitney-U-Test. Patients receiving an OLT during follow-up time were excluded in this analysis (N = 18). Values in the table are medians. Significant results are written in bold and asterisked (**, ***). Essential amino acids are printed in italics.

	Deceased patients (N = 33)	Surviving patients (N = 115)
Age [years]	58.62	57.70
Female	13 / 33 (39.4%)	40 / 115 (34.8%)
Male	20 / 33 (60.6%)	75 / 115 (65.2%)
MELD score	17.01	10.24***
MELD-Na score	19.88 (N = 31)	12.00***
*Phenylalanine* [μmol/l]	87.90	71.18**
Tyrosine [μmol/l]	108.94	94.00*
*Valine* [μmol/l]	131.40	148.77***
BCAA [μmol/l]	238.80	256.60*
AAA [μmol/l]	209.91	181.67**
Fischer’s ratio (BCAA / AAA)	1.12	1.37***
BTR (BCAA / Tyr)	2.13	2.75**
VPR (Val / Phe)	1.44	1.91***

Val: Valine. Phe: Phenylalanine. Tyr: Tyrosine.

BCAA: Branched-chain amino acids, sum of valine, leucine and isoleucine.

AAA: Aromatic amino acids, sum of phenylalanine, tyrosine and tryptophan.

**Fig 1:**
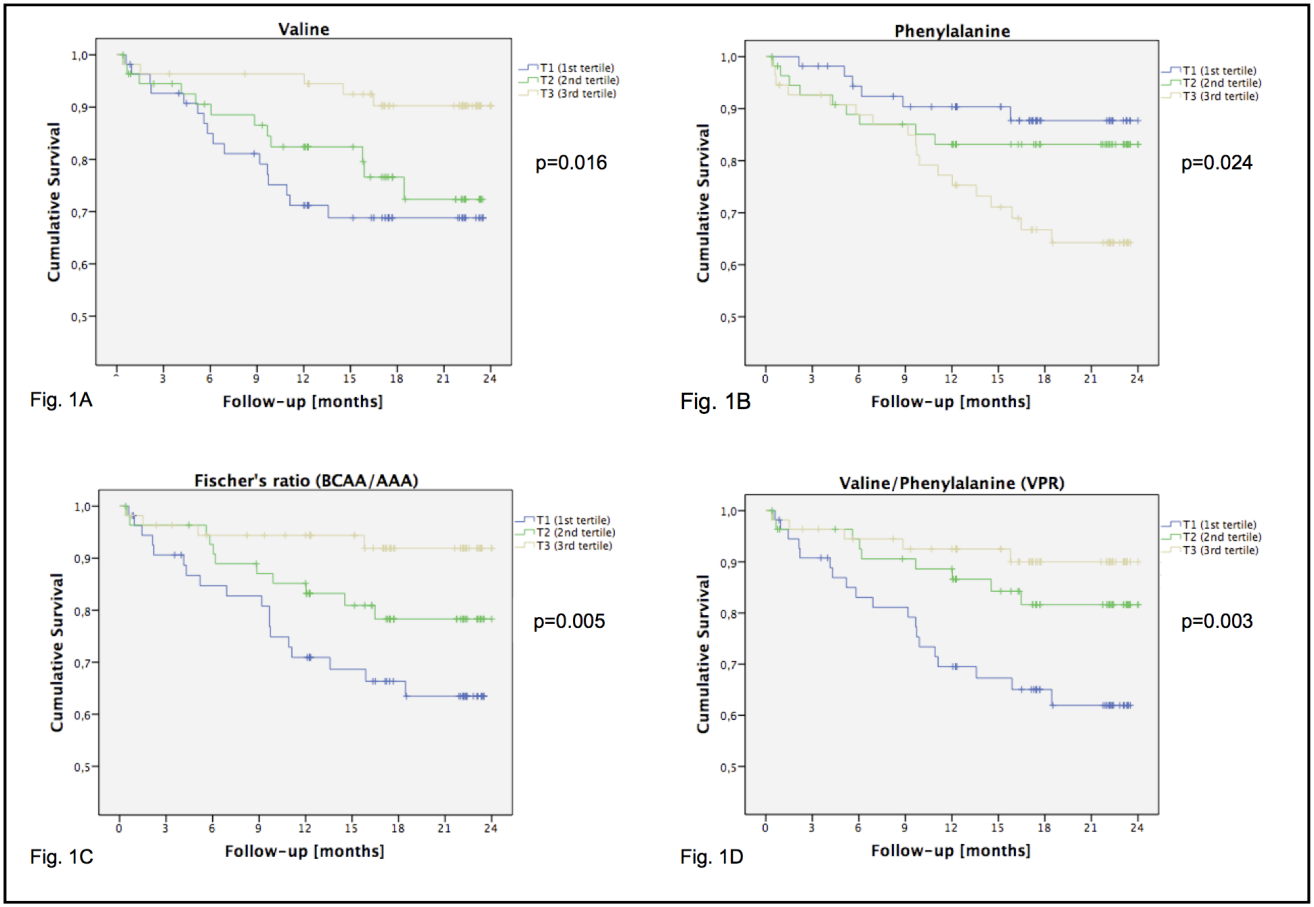
Cumulative survival according to Kaplan-Meier for chosen variables (N = 166). Presented p-values are from global Log-Rank tests. Maximum follow-up was 24 months. T1 (blue): group below the 33rd percentile. T2 (green): group within the 33rd and 67th percentile. T3 (yellow): group above the 67th percentile. BCAA: Branched-chain amino acids, sum of valine, leucine and isoleucine. AAA: Aromatic amino acids, sum of phenylalanine, tyrosine and tryptophan.

For further illustration, we performed Log-Rank testing (S1 Table). The tertiles discriminated survival rates significantly for the amino acids valine (p<0.05, Fig 1A) and phenylalanine (p<0.05, Fig 1B), but not for tyrosine (p = 0.20). For valine, data revealed a higher risk of death for group T1 to T3 (HR = 3.89; 95% CI, 1.42 to 10.65; p = 0.008) and for T2 to T3 (HR = 3.02; 95% CI, 1.06 to 8.61; p = 0.039), whereas the hazard ratio for T1 to T2 was not significant (p = 0.424). For phenylalanine, analysis led to a higher risk of death for group T3 to T1 (HR = 3.03; 95% CI, 1.20 to 7.63; p = 0.019), whereas hazard ratios for T3 to T2 (p = 0.079) and T2 to T1 (p = 0.411) were not discriminative.

For the calculated amino acid constellations, tertiles discriminated the risk of death significantly for Fischer’s ratio (p<0.01, Fig 1C) and for VPR (p<0.01, Fig 1D), whereas for BCAA, AAA and BTR results were not significant. Regarding Fischer’s ratio, data revealed a higher risk of death for group T1 to T3 (HR = 5.00; 95% CI, 1.69 to 14.78; p = 0.004), whereas hazard ratios between T1 to T2 (p = 0.117) and T2 to T3 (p = 0.081) were not significant. For VPR, analysis led to a higher risk of death for group T1 to T2 (HR = 2.32; 95% CI, 1.05 to 5.14; p = 0.037) and T1 to T3 (HR = 4.22; 95% CI, 1.57 to 11.30; p = 0.004), results for T2 to T3 did not differ significantly (p = 0.278).

### Analysis in context to MELD score

Furthermore, we studied the association between the amino acid parameters and established predictors of short-term mortality.

There were significantly different distributions of the plasma amino acids between groups with low MELD_6-9_ and moderate MELD_10-19_ scores (p<0.01, Table 5).

**Table 5:** Description of the analyzed cohort divided in three groups according to MELD score ranges 6 to 9, 10 to 19 and 20 to 40. Values in the table are medians. Distributions in group MELD_6-9_ compared to MELD_10-19_ and MELD_10-19_ compared to MELD_20-40_ were analyzed using Mann-Whitney-U-Test. Significant results are written in bold and asterisked (*, **, ***).

	MELD_6-9_, N = 53	MELD_10-19_, N = 90	MELD_20-40_, N = 23
MELD score	7.99	13.03***	22.80***
MELD-Na score	8.80	15.04*** (N = 89)	26.20*** (N = 21)
Creatinine [μmol/l]	74	82**	94
Bilirubin [μmol/l]	14.9	31.7***	121.0***
INR	1.1	1.3***	2.0***
Sodium [mmol/l]	139.0	137.0*** (N = 89)	134.5* (N = 21)
Phenylalanine [μmol/l]	65.63	77.44**	82.93
Valine [μmol/l]	165.87	136.30***	118.46
BCAA [μmol/l]	285.14	239.94***	213.05
AAA [μmol/l]	156.89	191.57**	210.39
Fischer’s ratio (BCAA / AAA)	1.87	1.24***	1.01**
BTR (BCAA / Tyr)	3.68	2.31***	1.90*
VPR (Val / Phe)	2.62	1.76***	1.31***

Val: Valine. Phe: Phenylalanine. Tyr: Tyrosine.

BCAA: Branched-chain amino acids, sum of valine, leucine and isoleucine.

AAA: Aromatic amino acids, sum of phenylalanine, tyrosine and tryptophan.

**Fig 2:**
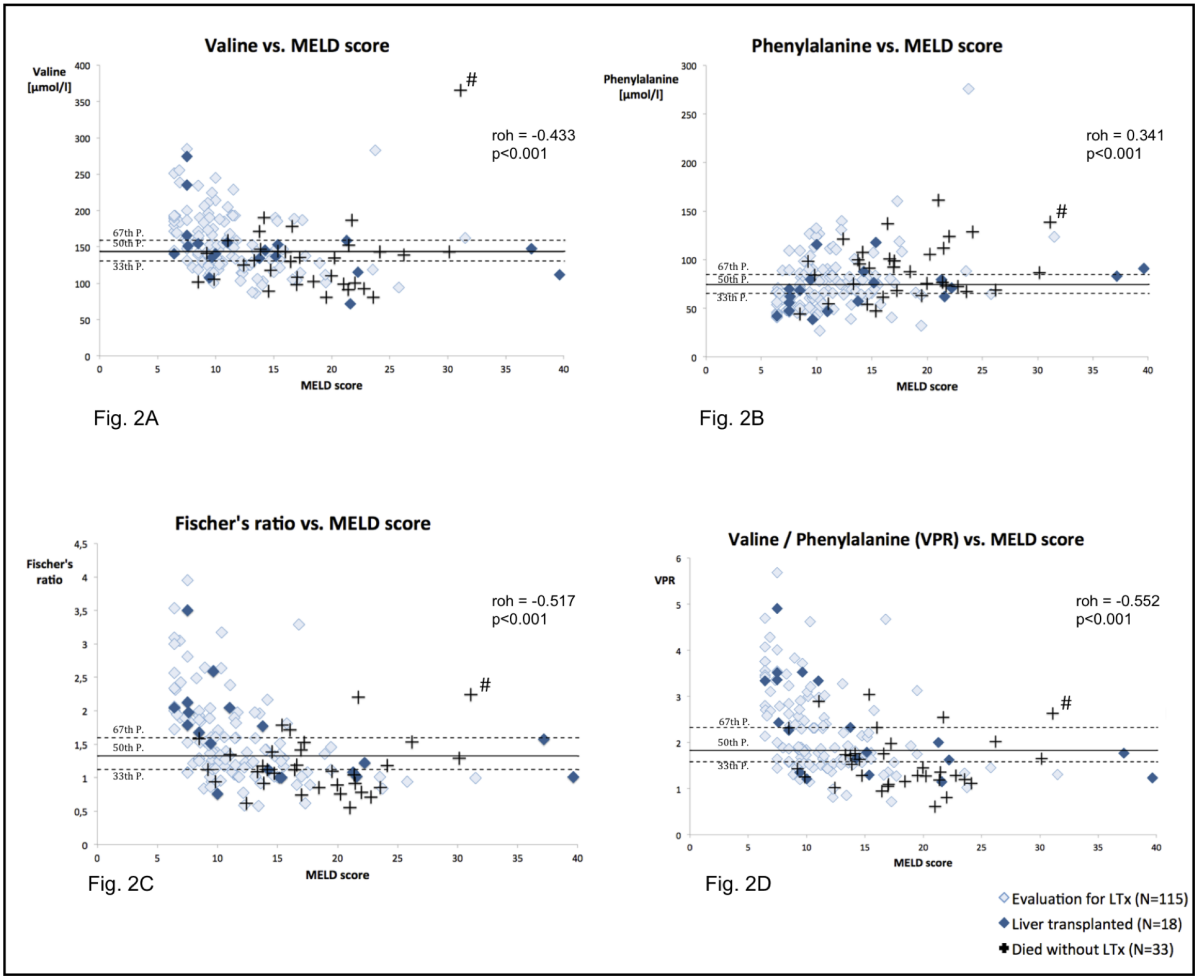
Correlation diagrams for chosen metabolites versus unrounded MELD scores (N = 166). LTx: liver transplantation. roh: Spearman’s roh coefficient. #: Patient who received Fresubin^®^ Hepa before blood taking at Intensive Care Unit.

MELD score was significantly positively correlated with arginine (p<0.01), aspartic acid (p<0.05), citrulline (p<0.05), methionine (p<0.001), phenylalanine (p<0.001, Fig 2B), proline (p<0.05) and tyrosine (p<0.001). Significant negative correlations were observed between MELD score and valine (p<0.001, Fig 2A) and the sum of leucine and isoleucine (p<0.05). Strongest associated branched-chain amino acid was valine (Spearman’s roh = -0.433) and strongest associated aromatic amino acid was phenylalanine (roh = 0.341).

BCAA, Fischer’s ratio (Fig 2C), BTR and VPR (Fig 2D) were all significantly negatively correlated with MELD score (p<0.001), whereas AAA showed a positive correlation (p<0.001). Regarding to rank correlation coefficients, only VPR and Fischer’s ratio were superior to valine alone.

Results for MELD-Na score correlation analysis were comparable (S2 Table).

Although there was a strong correlation between amino acid constellations and MELD score, we were able to show an additional prognostic value for valine plasma concentrations (p = 0.007; odds ratio = 0.78 per 10% change; 95% CI, 0.65 to 0.94) but not for phenylalanine (p = 0.803) when analyzed in addition to MELD score for prediction of one-year mortality.

Interestingly, we identified one obvious outlier in all correlations. This patient received Fresubin^®^ Hepa, a BCAA enriched solution, right before blood taking at the Intensive Care Unit (Fig 2, #).

## Discussion

The results of our study demonstrate that plasma concentrations of valine, phenylalanine and tyrosine are associated with survival in patients suffering from end-stage liver disease. Especially low concentrations of valine and the valine-containing ratio of branched-chain amino acids to aromatic amino acids (Fischer’s ratio) are prognostically unfavorable. In particular, a low ratio of valine to phenylalanine (VPR) results in a considerably increased mortality. VPR, Fischer’s ratio and valine showed a strong correlation with the three-month mortality predictors MELD and MELD-Na score.

Alterations in plasma levels of amino acids in liver cirrhosis are believed to play a role in causing hepatic encephalopathy (HE) [1]. HE is a serious complication in patients with end-stage liver disease and associated with an increased mortality. For this reason, it was part of the Child-Pugh Turcotte classification formerly used to decide on liver allocation [10]. Aromatic amino acid levels (AAA) increase in progression of cirrhosis, presumably due to shunt effects and missing uptake from hepatocytes [21]. Furthermore, detoxification of ammonia in the liver is impaired in patients with end-stage liver disease which leads to permanent hyperammonemia in blood. As an important compensatory mechanism, breakdown of BCAA storages (valine, leucine and isoleucine) in skeletal muscle is described to catabolize ammonia in the body [1].

BCAA and AAA share the L1 amino acid transporter of the blood-brain barrier. This imbalance in concentrations of BCAA to AAA leads to an increased influx of AAA that influences neurotransmitter synthesis. The resulting “false neurotransmitters” phenylethanolamine and octopamine affect the physiological neurotransmitters, especially noradrenaline, through competitive inhibition and further promote HE in this context. This potential mechanism was already published in 1971 by Fischer et al. [3] and the ratio of BCAA to AAA was named Fischer’s ratio. The false neurotransmitter hypothesis is discussed as a variable in the multifactorial pathophysiology of HE.

Another prognostically unfavorable factor in patients with liver cirrhosis is sarcopenia. As described, catabolism of BCAA compensates ammonia detoxification for the impaired liver to a certain extent. This constant consumption leads to low BCAA plasma concentrations and consequently sarcopenia [22,23]. Montano-Loza et al. presented data showing that sarcopenia, measured with cross-sectional imaging methods (CT scan or MRI), is associated with an increased mortality [23,24]. Another study showed that in cirrhotic patients with ascites, psoas muscle thickness (PMTH) could predict one-year mortality independent of MELD, MELD-Na and Child-Pugh-Turcotte score [25].

However, although plausible, in our study we could not prove the relationship between muscle mass and low levels of plasma BCAA because imaging data was not available.

Former studies also demonstrated that branched-chain amino acids influence mTOR (mammalian target of rapamycin) pathway which could lead to complex consequences [26,27]. Kakazu et al. [28] published that especially a valine decrease, mediated through mTOR pathway, has a negative impact on maturation and function of monocyte-derived dendritic cells in patients with hepatitis C virus related cirrhosis. Consequently, the immunologic response may be restricted in these patients leading to complications such as spontaneous bacterial peritonitis or sepsis. A decrease of leucine and isoleucine, however, did not affect maturation in their study. The sum parameter of leucine and isoleucine correlated significantly with MELD and MELD-Na score in our cohort, but did not have a significant impact on survival.

Although the MELD score is a well evaluated predictor of mortality, it has been shown that it does not reflect these relevant complications in end-stage liver disease adequately. In our study we were able to show a slightly more precise estimation of one-year mortality when including valine plasma concentrations to MELD score.

Based on the fact that the amino acids valine and phenylalanine belong to the essential amino acids, our data might lead to therapeutic implications. Previous studies examined the benefit of BCAA intake in cirrhosis [29] or in case of hepatic encephalopathy in particular [30,31,32]. Although results differ depending on the initial status of the analyzed patients, recently published clinical practice guidelines from AASLD and EASL recommend an oral BCAA-enriched diet as an alternative or additional agent to conventional therapy with lactulose [33]. Randomized controlled studies evaluating beneficial effects of an AAA restriction may be important for an optimal management of patients with end-stage liver disease.

In this context, the monitoring of amino acid concentrations in plasma could be a valuable instrument for the identification of patients with low BCAA or high AAA levels to create individual diet plans and to verify the treatment adherence, but since now, appropriate studies are lacking.

Since introduction of the MELD scoring system, waiting list mortality has decreased whereas postoperative mortality has increased [34]. Therefore, a debate about ethical principles and fairness of liver allocation is ongoing, also including the therapy success after organ transplantation [35]. Previous studies showed that preoperative sarcopenia and grade 3–4 of HE both are predictors of mortality after liver transplantation independent of MELD score [36,37]. Although we were not able to prove the relevance of amino acids and derivatives for predicting postoperative survival in our study, there is evidence in literature implicating that low values of BCAA to tyrosine ratio (BTR) might be unfavorable for patients who received a living donor liver transplant [38]. Hence, we speculate that measurement of metabolites has a prognostic value and could be a therapeutic target to improve the outcome after liver transplantation.

Our study analysis led to significant results concerning amino acids and prognosis in cirrhosis, nevertheless some limitations need to be considered. Data concerning muscular mass and HE were not available which prevent linkage between amino acid concentrations and phenotypes in our cohort. Though, objective and exact determination in particular for HE is difficult to assess. Secondly, the study subjects were not necessarily at fasting which might influence the metabolite measurements. One patient received Fresubin^®^ Hepa, whereas we found no evidence for therapeutic BCAA intake in any other patient. Furthermore, the heterogeneous study population with the presence of hepatocellular carcinoma (HCC) as a comorbidity in cirrhosis in 25 patients (15.1%). Although exclusion of those patients from analysis did not show a significant difference (data not shown), there is evidence showing that branched-chain amino acid metabolism differs in cirrhosis with and without presence of HCC and BCAA to tyrosine ratio (BTR) had a prognostic value for these patients [39]. In our study cohort, BTR also was predictive but inferior to Fischer’s ratio and VPR.

Plasma amino acid constellations are promising additional biomarkers for identification of patients with an increased risk of mortality in end-stage liver disease. Branched-chain amino acids, especially valine, and aromatic amino acids seem to participate in profound metabolic changes contributing to mortality. Our approach is considered to serve as a basis for prospective intervention studies. The identified panel has the potential to improve prognosis for patients with end-stage liver disease on the waiting list and after an orthotopic liver transplantation.

## Supporting Information

S1 Table Results of global Log-Rank tests. Tertiles were used for group division.
(DOCX)

S2 Table Spearman’s rank correlation coefficients. (DOCX)

## References

[1] Holecek M. Ammonia and amino acid profiles in liver cirrhosis: effects of variables leading to hepatic encephalopathy. Nutr Burbank Los Angel Cty Calif. 2015;31: 14–20. 10.1016/j.nut.2014.03.016 25220875

[2] Plauth M, Schütz T. Branched-chain amino acids in liver disease: new aspects of long known phenomena. Curr Opin Clin Nutr Metab Care. 2011;14: 61–66. 10.1097/MCO.0b013e3283413726 21088568

[3] Fischer JE, Baldessarini RJ. False neurotransmitters and hepatic failure. Lancet. 1971;2: 75–80. 4103986 10.1016/s0140-6736(71)92048-4

[4] Fischer JE, Yoshimura N, Aguirre A, James JH, Cummings MG, Abel RM, et al Plasma amino acids in patients with hepatic encephalopathy. Effects of amino acid infusions. Am J Surg. 1974;127: 40–47. 4808685 10.1016/0002-9610(74)90009-9

[5] Kamath PS, Wiesner RH, Malinchoc M, Kremers W, Therneau TM, Kosberg CL, et al A model to predict survival in patients with end-stage liver disease. Hepatol Baltim Md. 2001;33: 464–470. 10.1053/jhep.2001.22172 11172350

[6] Kamath PS, Kim WR, Advanced Liver Disease Study Group. The model for end-stage liver disease (MELD). Hepatol Baltim Md. 2007;45: 797–805. 10.1002/hep.21563 17326206

[7] Malinchoc M, Kamath PS, Gordon FD, Peine CJ, Rank J, ter Borg PC. A model to predict poor survival in patients undergoing transjugular intrahepatic portosystemic shunts. Hepatol Baltim Md. 2000;31: 864–871. 10.1053/he.2000.5852 10733541

[8] Cholongitas E, Germani G, Burroughs AK. Prioritization for liver transplantation. Nat Rev Gastroenterol Hepatol. 2010;7: 659–668. 10.1038/nrgastro.2010.169 21045793

[9] Asrani SK, Kim WR. Model for end-stage liver disease: end of the first decade. Clin Liver Dis. 2011;15: 685–698. 10.1016/j.cld.2011.08.009 22032523 PMC3564596

[10] Yoo HY, Edwin D, Thuluvath PJ. Relationship of the model for end-stage liver disease (MELD) scale to hepatic encephalopathy, as defined by electroencephalography and neuropsychometric testing, and ascites. Am J Gastroenterol. 2003;98: 1395–1399. 10.1111/j.1572-0241.2003.07466.x 12818287

[11] Kachaamy T, Bajaj JS, Heuman DM. Muscle and mortality in cirrhosis. Clin Gastroenterol Hepatol Off Clin Pract J Am Gastroenterol Assoc. 2012;10: 100–102. 10.1016/j.cgh.2011.11.002 22079850

[12] Ney M, Abraldes JG, Ma M, Belland D, Harvey A, Robbins S, et al Insufficient Protein Intake Is Associated With Increased Mortality in 630 Patients With Cirrhosis Awaiting Liver Transplantation. Nutr Clin Pract Off Publ Am Soc Parenter Enter Nutr. 2015; 10.1177/0884533614567716 25667232

[13] Schwabl P, Bucsics T, Soucek K, Mandorfer M, Bota S, Blacky A, et al Risk factors for development of spontaneous bacterial peritonitis and subsequent mortality in cirrhotic patients with ascites. Liver Int Off J Int Assoc Study Liver. 2015; 10.1111/liv.12795 25644943

[14] Olde Damink SWM, Jalan R, Deutz NEP, Dejong CHC, Redhead DN, Hynd P, et al Isoleucine infusion during “simulated” upper gastrointestinal bleeding improves liver and muscle protein synthesis in cirrhotic patients. Hepatol Baltim Md. 2007;45: 560–568. 10.1002/hep.21463 17326149

[15] Ginès P, Guevara M. Hyponatremia in cirrhosis: pathogenesis, clinical significance, and management. Hepatol Baltim Md. 2008;48: 1002–1010. 10.1002/hep.22418 18671303

[16] Kim WR, Biggins SW, Kremers WK, Wiesner RH, Kamath PS, Benson JT, et al Hyponatremia and Mortality among Patients on the Liver-Transplant Waiting List. N Engl J Med. 2008;359: 1018–1026. 10.1056/NEJMoa0801209 18768945 PMC4374557

[17] Wedd J, Bambha KM, Stotts M, Laskey H, Colmenero J, Gralla J, et al Stage of cirrhosis predicts the risk of liver-related death in patients with low Model for End-Stage Liver Disease scores and cirrhosis awaiting liver transplantation. Liver Transplant Off Publ Am Assoc Study Liver Dis Int Liver Transplant Soc. 2014;20: 1193–1201. 10.1002/lt.23929 PMC4177271 24916539

[18] Ceglarek U, Müller P, Stach B, Bührdel P, Thiery J, Kiess W. Validation of the Phenylalanine/Tyrosine Ratio Determined by Tandem Mass Spectrometry: Sensitive Newborn Screening for Phenylketonuria. Clin Chem Lab Med. 2002;40: 693–697. 12241016 10.1515/CCLM.2002.119

[19] Brauer R, Leichtle AB, Fiedler GM, Thiery J, Ceglarek U. Preanalytical standardization of amino acid and acylcarnitine metabolite profiling in human blood using tandem mass spectrometry. Metabolomics. 2010;7: 344–352. 10.1007/s11306-010-0256-1

[20] MELD/PELD Calculator Documentation [Internet]. 28 1 2009 Available: https://www.unos.org/wp-content/uploads/unos/MELD_PELD_Calculator_Documentation.pdf

[21] Dejong CHC, van de Poll MCG, Soeters PB, Jalan R, Olde Damink SWM. Aromatic amino acid metabolism during liver failure. J Nutr. 2007;137: 1579S–1585S; discussion 1597S–1598S. 17513430 10.1093/jn/137.6.1579S

[22] Holecek M. Branched-chain amino acids and ammonia metabolism in liver disease: therapeutic implications. Nutr Burbank Los Angel Cty Calif. 2013;29: 1186–1191. 10.1016/j.nut.2013.01.022 23756281

[23] Montano-Loza AJ, Meza-Junco J, Prado CMM, Lieffers JR, Baracos VE, Bain VG, et al Muscle wasting is associated with mortality in patients with cirrhosis. Clin Gastroenterol Hepatol Off Clin Pract J Am Gastroenterol Assoc. 2012;10: 166–173, 173.e1 10.1016/j.cgh.2011.08.028 21893129

[24] Montano-Loza AJ. Muscle wasting: a nutritional criterion to prioritize patients for liver transplantation. Curr Opin Clin Nutr Metab Care. 2014;17: 219–225. 10.1097/MCO.0000000000000046 24613858

[25] Kim TY, Kim MY, Sohn JH, Kim SM, Ryu JA, Lim S, et al Sarcopenia as a useful predictor for long-term mortality in cirrhotic patients with ascites. J Korean Med Sci. 2014;29: 1253–1259. 10.3346/jkms.2014.29.9.1253 25246744 PMC4168179

[26] Kakazu E, Kondo Y, Kogure T, Ninomiya M, Kimura O, Ueno Y, et al Plasma amino acids imbalance in cirrhotic patients disturbs the tricarboxylic acid cycle of dendritic cell. Sci Rep. 2013;3: 3459 10.1038/srep03459 24322372 PMC3857572

[27] Kakazu E, Ueno Y, Kondo Y, Fukushima K, Shiina M, Inoue J, et al Branched chain amino acids enhance the maturation and function of myeloid dendritic cells ex vivo in patients with advanced cirrhosis. Hepatol Baltim Md. 2009;50: 1936–1945. 10.1002/hep.23248 19885880

[28] Kakazu E, Kanno N, Ueno Y, Shimosegawa T. Extracellular branched-chain amino acids, especially valine, regulate maturation and function of monocyte-derived dendritic cells. J Immunol Baltim Md 1950. 2007;179: 7137–7146. 10.4049/jimmunol.179.10.7137 17982106

[29] Tsien C, Davuluri G, Singh D, Allawy A, Ten Have GAM, Thapaliya S, et al Metabolic and molecular responses to leucine-enriched branched chain amino acid supplementation in the skeletal muscle of alcoholic cirrhosis. Hepatology. 2015;61: 2018–2029. 10.1002/hep.27717 25613922 PMC4441611

[30] Gluud LL, Dam G, Borre M, Les I, Cordoba J, Marchesini G, et al Oral branched-chain amino acids have a beneficial effect on manifestations of hepatic encephalopathy in a systematic review with meta-analyses of randomized controlled trials. J Nutr. 2013;143: 1263–1268. 10.3945/jn.113.174375 23739310

[31] Kawaguchi T, Izumi N, Charlton MR, Sata M. Branched-chain amino acids as pharmacological nutrients in chronic liver disease. Hepatol Baltim Md. 2011;54: 1063–1070. 10.1002/hep.24412 21563202

[32] Kawamura E, Habu D, Morikawa H, Enomoto M, Kawabe J, Tamori A, et al A randomized pilot trial of oral branched-chain amino acids in early cirrhosis: validation using prognostic markers for pre-liver transplant status. Liver Transplant Off Publ Am Assoc Study Liver Dis Int Liver Transplant Soc. 2009;15: 790–797. 10.1002/lt.21758 19562716

[33] American Association for the Study of Liver Diseases, European Association for the Study of the Liver. Hepatic encephalopathy in chronic liver disease: 2014 practice guideline by the European Association for the Study of the Liver and the American Association for the Study of Liver Diseases. J Hepatol. 2014;61: 642–659. 10.1016/j.jhep.2014.05.042 25015420

[34] Weismüller TJ, Negm A, Becker T, Barg-Hock H, Klempnauer J, Manns MP, et al The introduction of MELD-based organ allocation impacts 3-month survival after liver transplantation by influencing pretransplant patient characteristics. Transpl Int Off J Eur Soc Organ Transplant. 2009;22: 970–978. 10.1111/j.1432-2277.2009.00915.x 19619170

[35] Bobbert M, Ganten TM. Liver allocation: urgency of need or prospect of success? Ethical considerations. Clin Transplant. 2013;27 Suppl 25: 34–39. 10.1111/ctr.12154 23909500

[36] Englesbe MJ, Patel SP, He K, Lynch RJ, Schaubel DE, Harbaugh C, et al Sarcopenia and mortality after liver transplantation. J Am Coll Surg. 2010;211: 271–278. 10.1016/j.jamcollsurg.2010.03.039 20670867 PMC2914324

[37] Wong RJ, Aguilar M, Gish RG, Cheung R, Ahmed A. The impact of Pre-transplant hepatic encephalopathy on survival following liver transplantation. Liver Transpl. 2015; n/a–n/a. 10.1002/lt.24153 25902933

[38] Hamaguchi Y, Kaido T, Okumura S, Fujimoto Y, Ogawa K, Mori A, et al Impact of quality as well as quantity of skeletal muscle on outcomes after liver transplantation. Liver Transplant Off Publ Am Assoc Study Liver Dis Int Liver Transplant Soc. 2014;20: 1413–1419. 10.1002/lt.23970 25088484

[39] Kawaguchi T, Shiraishi K, Ito T, Suzuki K, Koreeda C, Ohtake T, et al Branched-chain amino acids prevent hepatocarcinogenesis and prolong survival of patients with cirrhosis. Clin Gastroenterol Hepatol Off Clin Pract J Am Gastroenterol Assoc. 2014;12: 1012–1018.e1. 10.1016/j.cgh.2013.08.050 24036055

