# Supplementary material for: Plasma Amino Acid Concentrations Predict Mortality in Patients with End-Stage Liver Disease
Source: PLoS One. 2016 Jul 13;11(7):e0159205. doi: 10.1371/journal.pone.0159205 (PMC4943589; doi:10.1371/journal.pone.0159205)
Supplement: S1 Table — Tertiles were used for group division. (DOCX) [file pone.0159205.s001.docx]

| **S1 Table.** Results of global Log-Rank tests. Tertiles were used for group division. Significant results are written in bold and asterisked. Essential amino acids are printed in italics. | | | | | |
| --- | --- | --- | --- | --- | --- |
|  |  | **Group T1**  <33.3rd percentile | **Group T2**  33.3rd - 66.7th percentile | **Group T3**  >66.7th percentile | **Log-Rank Test** p-value |
|  |  |  |  |  |  |
| *Phenylalanine* | Median | 53.90µmol/l | 74.57µmol/l | 107.30µmol/l | **0.024*** |
|  | Range. | 27.20 - 65.40µmol/l | 65.40 - 84.13µmol/l | 86.26 - 276.09µmol/l |  |
| Tyrosine | Median | 64.97µmol/l | 96.23µmol/l | 151.20µmol/l | 0.201 |
|  | Range. | 31.47 - 78.63µmol/l | 79.03 - 111.15µmol/l | 111.70 - 235.49µmol/l |  |
| *Valine* | Median | 111.47µmol/l | 143.71µmol/l | 190.00µmol/l | **0.016*** |
|  | Range. | 71.70 - 130.67µmol/l | 130.72 - 158.80µmol/l | 159.92 - 365.60µmol/l |  |
|  |  |  |  |  |  |
| BCAA | Median | 195.87µmol/l | 254.68µmol/l | 342.27µmol/l | 0.151 |
|  | Range. | 157.00 - 224.93µmol/l | 225.84 - 282.77µmol/l | 285.14 - 779.80µmol/l |  |
| AAA | Median | 131.49µmol/l | 184.76µmol/l | 262.70µmol/l | 0.131 |
|  | Range. | 81.40 - 159.03µmol/l | 160.52 - 210.07µmol/l | 210.39 - 538.26µmol/l |  |
| Fischer’s ratio  (BCAA / AAA) | Median | 0.99µmol/l | 1.33µmol/l | 2.04µmol/l | **0.005**** |
|  | Range. | 0.56 - 1.12µmol/l | 1.12 - 1.60µmol/l | 1.61 - 3.95µmol/l |  |
| BTR  (BCAA / Tyr) | Median | 1.78µmol/l | 2.68µmol/l | 4.10µmol/l | 0.054 |
|  | Range. | 0.98 - 2.08µmol/l | 2.09 - 3.20µmol/l | 3.25 - 9.37µmol/l |  |
| VPR  (Val / Phe) | Median | 1.30µmol/l | 1.83µmol/l | 3.10µmol/l | **0.003**** |
|  | Range. | 0.62 - 1.58µmol/l | 1.59 - 2.33µmol/l | 2.33 - 5.68µmol/l |  |
| Val: Valine. Phe: Phenylalanine. Tyr: Tyrosine.  BCAA: Branched-chain amino acids, sum of valine, leucine and isoleucine.  AAA: Aromatic amino acids, sum of phenylalanine, tyrosine and tryptophan. | | | | | |
