# Supplementary material for: Plasma Amino Acid Concentrations Predict Mortality in Patients with End-Stage Liver Disease
Source: PLoS One. 2016 Jul 13;11(7):e0159205. doi: 10.1371/journal.pone.0159205 (PMC4943589; doi:10.1371/journal.pone.0159205)
Supplement: S2 Table — (DOCX) [file pone.0159205.s002.docx]

| **S2 Table**. Spearman’s rank correlation coefficients. Significant results are annotated with asterisks (p<0.05*, p<0.01**, p<0.001***). Significant positive correlations are colored blue, in contrast significant negative correlations are colored red (the darker, the higher the level of significance). Furthermore level of significance p<0.001 is marked in bold numbers. Essential amino acids are printed in italics. | | | | | | |
| --- | --- | --- | --- | --- | --- | --- |
|  | **MELD**  **N=166** | **MELD-Na**  **N=163** | **Creatinine**  **N=166** | **Bilirubin**  **N=166** | **INR**  **N=166** | **Sodium**  **N=163** |
| Alanine | roh = -0.136 | roh = -0.156* | roh = 0.018 | roh = -0.098 | roh = -0.180* | roh = 0.142 |
| Arginine | roh = 0.231** | **roh = 0.236**** | roh = 0.051 | roh = 0.236** | roh = 0.199** | roh = -0.133 |
| Aspartic acid | roh = 0.191* | roh = 0.166* | roh = 0.071 | roh = 0.189* | roh = 0.167* | roh = -0.043 |
| Citrulline | roh = 0.185* | roh = 0.177* | **roh = 0.266***** | roh = 0.138 | roh = 0.135 | roh = -0.038 |
| Glutamic acid | roh = -0.079 | roh = -0.063 | roh = 0.083 | roh = -0.058 | roh = -0.176* | roh = 0.104 |
| Glycine | roh = 0.065 | roh = 0.050 | roh = 0.177* | roh = 0.048 | roh = 0.023 | roh = 0.029 |
| Histidine | roh = 0.138 | roh = 0.114 | roh = 0.109 | roh = 0.168* | roh = 0.093 | roh = -0.001 |
| *Leucine* and  *Isoleucine* | roh = -0.182* | roh = -0.180* | roh = 0.114 | roh = -0.154* | roh = -0.226** | roh = 0.143 |
| *Lysine* | roh = -0.071 | roh = -0.064 | roh = -0.007 | roh = 0.006 | roh = -0.121 | roh = 0.126 |
| *Methionine* | **roh = 0.341*****  (n=164) | **roh = 0.356*****  (n=161) | roh = 0.147  (n=164) | **roh = 0.381*****  (n=164) | **roh = 0.342*****  (n=164) | roh = -0.205**  (n=161) |
| Ornithine | roh = -0.013 | roh = -0.048 | roh = 0.048 | roh = 0.024 | roh = -0.051 | roh = 0.058 |
| *Phenylalanine* | **roh = 0.341***** | **roh = 0.346***** | roh = 0.180* | **roh = 0.318***** | **roh = 0.304***** | roh = -0.190* |
| Proline | roh = 0.172* | roh = 0.202** | roh = 0.143 | roh = 0.124 | roh = 0.103 | roh = -0.045 |
| Serine | roh = -0.122 | roh = -0.114 | roh = -0.057 | roh = -0.039 | roh = -0.149 | roh = 0.138 |
| *Threonine* | roh = 0.015 | roh = 0.032 | roh = -0.079 | roh = 0.063 | roh = 0.023 | roh = 0.080 |
| *Tryptophan* | roh = 0.050 | roh = -0.005 | roh = -0.063 | roh = 0.095 | roh = 0.019 | roh = 0.094 |
| Tyrosine | **roh = 0.325***** | **roh = 0.337***** | roh = -0.044 | **roh = 0.390***** | **roh = 0.327***** | roh = -0.139 |
| *Valine* | **roh = -0.433***** | **roh = -0.436***** | roh = -0.114 | **roh = -0.335***** | **roh = -0.431***** | **roh = 0.304***** |
|  |  |  |  |  |  |  |
| BCAA | **roh = -0.342***** | **roh = -0.343***** | roh = -0.003 | **roh = -0.270***** | **roh = -0.367***** | **roh = 0.249***** |
| AAA | **roh = 0.356***** | **roh = 0.364***** | roh = 0.040 | **roh = 0.390***** | **roh = 0.342***** | roh = -0.170* |
| Fischer’s ratio (BCAA / AAA) | **roh = -0.517***** | **roh = -0.517***** | roh = -0.013 | **roh = -0.503***** | **roh = -0.516***** | **roh = 0.309***** |
| BTR (BCAA / Tyr) | **roh = -0.472***** | **roh = -0.481***** | roh = 0.056 | **roh = -0.493***** | **roh = -0.479***** | **roh = 0.271***** |
| VPR (Val / Phe) | **roh = -0.552***** | **roh = -0.552***** | roh = -0.198* | **roh = -0.477***** | **roh = -0.511***** | **roh = 0.354***** |
| Val: Valine. Phe: Phenylalanine. Tyr: Tyrosine.  BCAA: Branched-chain amino acids, sum of valine, leucine and isoleucine.  AAA: Aromatic amino acids, sum of phenylalanine, tyrosine and tryptophan. | | | | | | |
